# Effects of Different Irrigation Regimes, Nitrogen Levels and Storage Conditions on Volatiles of ‘Gala’ Apple

**DOI:** 10.3390/molecules28186610

**Published:** 2023-09-14

**Authors:** Cláudia Tavares, Carolina Ferro Rodrigues, Elsa Gonçalves, Alexandra M. Machado, Luís Pedro, José Barroso, Anabela Maurício, Nuno Franco, Délio Raimundo, Valério Pita, Claudia Sánchez, Ana Cristina Figueiredo

**Affiliations:** 1 Centro de Estudos do Ambiente e do Mar (CESAM Ciências), Faculdade de Ciências da Universidade de Lisboa (FCUL), Biotecnologia Vegetal, DBV, C2, Campo Grande, 1749-016 Lisboa, Portugal; claudiasstavares@gmail.com (C.T.); carolina_ferro_rodrigues@hotmail.com (C.F.R.); evgoncalves@fc.ul.pt (E.G.); ampmachado@fc.ul.pt (A.M.M.); lmpedro@fc.ul.pt (L.P.); jmbarroso@fc.ul.pt (J.B.); 2 Frubaça, Cooperativa de Horto—Fruticultores, C.R.L. Lugar Acipreste Aptd. 12, 2460-471 Alcobaça, Portugal; abcnm@sapo.pt (A.M.); nunohsfranco@gmail.com (N.F.); 3 Campotec—Conservação e Transformação de Hortofrutícolas, SA. Estrada Nacional 9, 2560-393 Torres Vedras, Portugal; delio.raimundo@campotec.pt; 4 Cooperfrutas, Coop Produtores Fruta e Prod Horticolas de Alcobaca C.R.L., Quinta Freiras—Ponte Jardim, 2460-617 Aljubarrota, Portugal; valeriopita@cooperfrutas.pt; 5 Instituto Nacional de Investigação Agrária e Veterinária (INIAV), I.P., Polo Alcobaça, Estrada de Leiria, 2460-059 Alcobaça, Portugal; claudia.sanchez@iniav.pt; 6 GREEN-IT—Bioresources for Sustainability R&D Unit, Instituto de Tecnologia Química e Biológica António Xavier / Universidade Nova de Lisboa (ITQB NOVA), 2780-157 Oeiras, Portugal

**Keywords:** Maçã de Alcobaça, apple, Gala cultivar, SPME, volatiles, irrigation, nitrogen, storage

## Abstract

With a characteristic flavour and aroma, “Maçã de Alcobaça” are apples produced in the western region of the mainland of Portugal. Given the known influence of pre-harvest cultural techniques and post-harvest conservation methods on fruit quality, this work evaluated the effect of cultural factors and conservation methods on the volatile profile of ‘Gala’ apples. Tests were carried out during four seasons (2018 to 2021) in two ‘Gala’ apple orchards (F and S) maintained with different irrigation rates and nitrogen fertilisation [normal irrigation and normal nitrogen (Control, NINN), normal irrigation and excess nitrogen (NIEN), excess irrigation and normal nitrogen (EINN), excess irrigation and excess nitrogen (EIEN)], and under three storage conditions [Controlled Atmosphere + 1-methylcyclopropene (CA+1-MCP), Dynamic Controlled Atmosphere (DCA) and DCA+1-MCP]. The intact fruit volatiles were isolated by headspace solid–phase microextraction (HS–SPME) and analysed by Gas Chromatography with Flame Ionisation Detection and Gas Chromatography-Mass Spectrometry at harvest (T0) and after 8 months of storage (T8). HS–SPME volatiles from ‘Gala’ apples, obtained at T0 in control conditions, were characterised by *trans,trans*-α-farnesene dominance (36–69%), followed by hexyl acetate (5–23%) and hexyl hexanoate (3–9%). The four irrigation and nitrogen treatments did not evidence main changes in the apple volatile profile. Instead, storage conditions changed the ratio between compounds; previously undetected compounds attained high percentages and decreased the intensity of the dominant compounds in the control conditions. Although all storage conditions tested changed the volatile profile and emanation intensity, the effect was more accentuated in storage for 8 months with DCA+1-MCP.

## 1. Introduction

Alcobaça is a region on the west coast of mainland Portugal, with characteristic edaphoclimatic conditions. “Maçã de Alcobaça” are apples produced in this area belonging to nine different *Malus domestica* (Rosaceae) groups, with Protected Geographical Indication (PGI) [1]. Among these apples, the ‘Gala’ cultivar has a high demand in the national and international markets. 

The ‘Gala’ cultivar is one (Kidd’s D8) of a group of selections of Kidd’s Orange × Golden Delicious crosses that resulted from the original crosses of Cox Orange Pippin × Delicious from the New Zealand fruit grower J. H. Kidd back in the 1920s. Gala was later shown to be predisposed to colour mutations, and the red striped Royal Gala became favoured by consumers and was less prone to bruising than ‘Gala’ [2,3]. Several other ‘Gala’ crosses have been established since then, such as ‘Regal Gala’, ‘Imperial Gala’ and ‘Galaxy’ [2].

Previous studies have addressed the effect of pruning, thinning, fertilisation, rootstock and irrigation on ‘Gala’ apple yield and fruit size [4,5,6,7,8,9,10,11], as well as ‘Gala’ fruit quality and volatile emission after regular and controlled atmosphere storage, and/or fruit coating [3,12,13,14,15,16,17,18,19], the descriptive sensory analysis (DSA) of aroma changes during storage [20], the impact of ionising radiation on volatile production [21], or its α-farnesene and ester variability [22]. Nevertheless, to the best of our knowledge, there is no previous information on “Maçã de Alcobaça” ‘Gala’ volatiles, nor a combined evaluation of the effect of cultural practices and conservation method, on ‘Gala’ cultivar volatiles.

Despite the importance in slowing down the ripening effect, the storage of ‘Gala’ fruits under a controlled atmosphere (CA), in dynamic controlled atmosphere (DCA), under extreme low oxygen (ELO) associated with low O_2_ hysteresis (HY), or with the ethylene action inhibitor, 1-methylcyclopropene (1-MCP) has been shown to decrease volatile production [16,17,18,23,24,25,26,27] and other ‘Gala’ fruit attributes [28,29,30,31,32,33,34]. It is nevertheless important to mention that the degree of this decrease is dependent on the overall conditions of storage (CO_2_, O_2_, N_2_, temperature, period stored), as well as from the fruit cultivar and fruit maturity at harvest [16,17,18,23,24,25]. This effect has been mostly studied in apple ester emissions and in overall crop yield, and it has been suggested that the lower ester production on stored apples results from a lack of substrates, lower respiratory activity, changes in carboxy acid metabolism, and altered enzymatic activity [17,23,35,36].

Integrated in a wider goal of long-term preservation of “Maçã de Alcobaça” quality with zero residue in terms of post-harvest product application, the aim of the present study was to determine the volatile profile of ‘Gala’ apples and to assess the effect of cultural practices (irrigation amount and nitrogen fertilisation) and of storage, for 8 months, under different conservation methods [Controlled Atmosphere + 1-methylcyclopropene (CA + 1-MCP), Dynamic Controlled Atmosphere (DCA) and DCA + 1-MCP], on “Maçã de Alcobaça” ‘Gala’ volatiles.

## 2. Results

### 2.1. Apple Volatiles at Harvest Time (T0) from Orchards Plots Grown under Control Conditions

**Figure 1:**
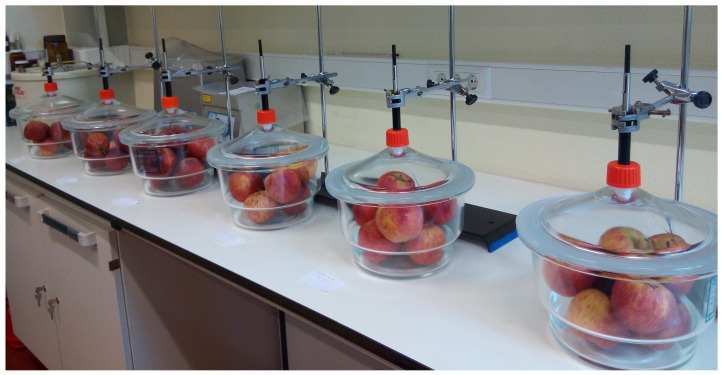
HS-SPME collection of volatiles from pooled samples of 7 ‘Gala’ apples (“Maçã de Alcobaça”) per desiccator at room temperature.

In total, 7 pooled samples of apples were obtained, at harvest (T0), from plots grown under regular irrigation and nitrogen fertilisation (Control), during the four-year study (1 pooled sample per orchard per year, except in the last year, each with ≈ 30–32 apples) (Table 1). The volatiles collected from intact ‘Gala’ apples, by headspace solid–phase microextraction (HS–SPME) (Figure 1), were complex mixtures in which up to forty components were identified. The isolated volatiles are listed in Table 2, following their elution order on the DB-1 column, and arranged according to the lowest and highest percentages found for each component in the four-year study. Compounds were also grouped according to the main biosynthetic pathway in terpenes (ex. farnesene), phenylpropanoids (estragole) and others resulting from the acetate pathway (ex. esters).

**Figure 2:**
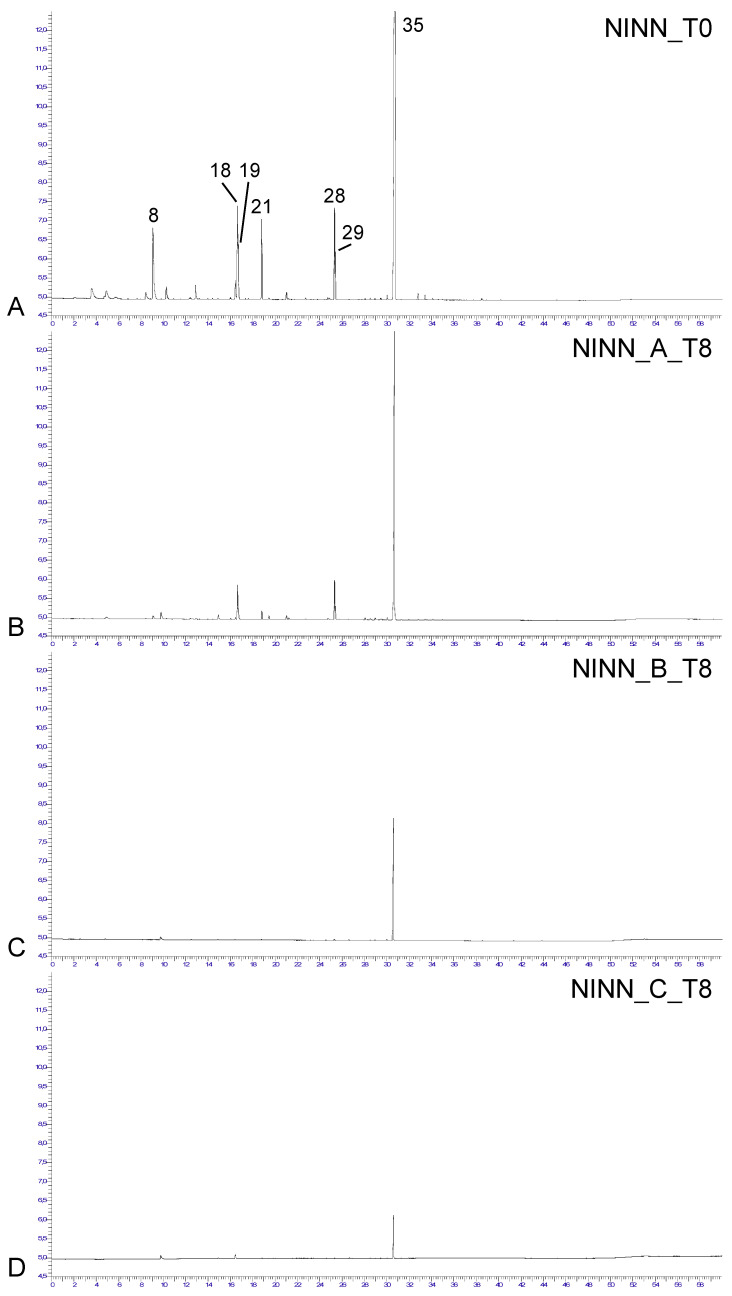
Representative HS-SPME-GC-FID profiles of volatiles from pooled samples of “Maçã de Alcobaça” ‘Gala’ apples at T0 (**A**). grown under control conditions, with regular irrigation and nitrogen fertilisation (NINN), and at T8, after storage, for 8 months, under different conservation methods (**B**). CA + 1-MCP (A). (**C**). DCA (B). (**D**). DCA + 1-MCP (C). Total run time: 61 min. For abbreviations, vide Table 1. For peak number correspondence, vide Table 2.

The characteristic volatile profile of intact “Maçã de Alcobaça” ‘Gala’ apples at harvest time (T0) from orchards grown under control conditions was dominated by *trans,trans*-α-farnesene (36–69%) (Table 2, Figure 2). Other components also attained high relative amounts (≥5% at least once during the four-year study), such as hexyl acetate (5–23%), hexyl hexanoate (3–9%), 2-methyl butyric acid hexyl ester (3–6%), and butyl acetate, butyl hexanoate, hexyl butanoate and butyl octanoate (all 2–6%).

### 2.2. Apple Volatiles at T0 and T8 from Orchards Plots Grown under All Conditions

Overall, 80 HS-SPME pooled apple samples of the four irrigation and fertilisation treatments and storage conditions were evaluated (Table 1). The relative amounts of all the identified components are listed in Table 3, arranged according to the lowest and the highest percentages found for each component in the two main clusters, and subclusters, defined by agglomerative cluster analysis, based on the average composition of each pooled apple sample volatiles.

**Figure 3:**
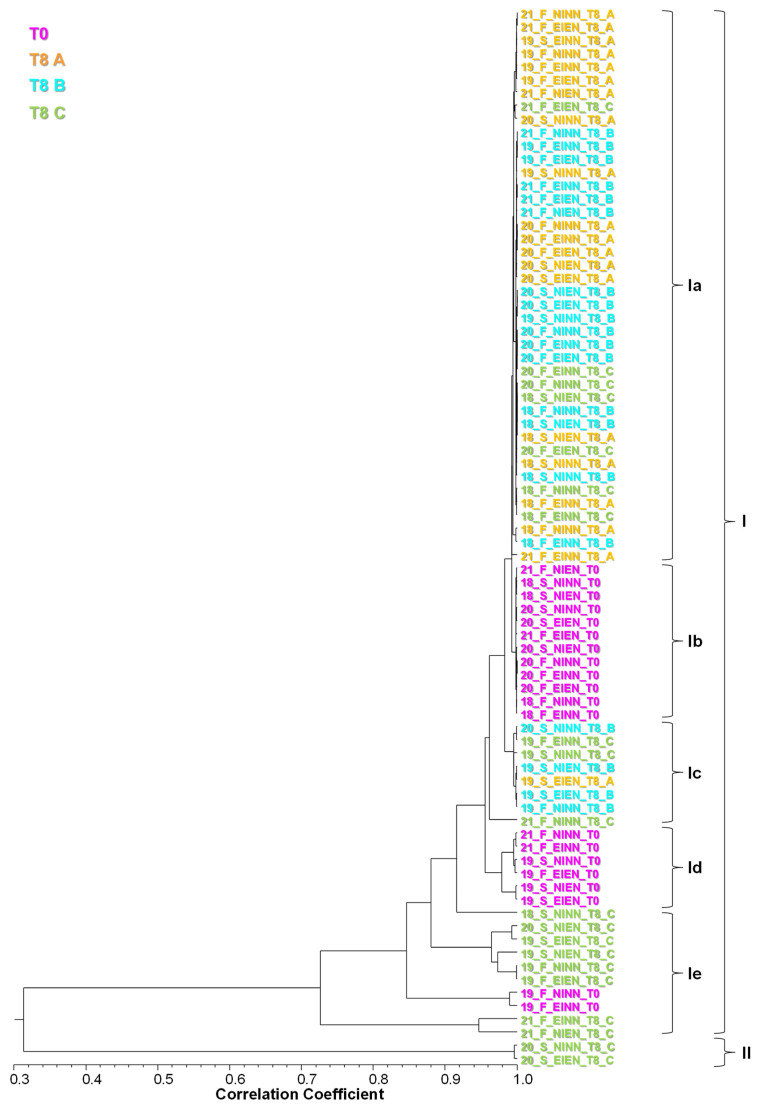
Dendrogram obtained by cluster analysis of the percentage composition of the HS-SPME volatiles isolated from apple fruits, based on correlation and using the unweighted pair-group method with arithmetic average (UPGMA). In sample codes, first numbers stand for collection year, followed by code letters referring to orchard and treatment condition as detailed in Table 1, if tested after harvesting (T0) or after 8 months (T8) of storage, under CA+1-MCP (A), DCA (B) and DCA+1-MCP (C). Cluster distribution is evidenced by the colour codes.

Cluster analysis showed two main clusters, I and II, with low correlation (Scorr ≤ 0.32; Figure 3, Table 3). Cluster I included 78 (97.5%) of the 80 pooled samples analysed and cluster II included the remaining 2 (2.5%) samples.

Cluster I included several subclusters with diverse degrees of correlation. Subclusters Ia to Id were all highly correlated (Scorr ≥ 0.94). Despite still having a high correlation (Scorr ≥ 0.72), subcluster Ie included several less correlated samples (Figure 3, Table 3).

Considering the volatiles from apples grown under control conditions and harvested at T0 as reference, the main differences that determined the observed clustering were due to changes in the relative amounts of some components and the presence, in variable amounts in T8 samples, of a compound that was not detected in the T0 samples, 2-ethyl-1-hexanol. Its presence in a very high percentage in two samples (65% and 71%) determined cluster II, as this compound was not detected or was <39% in the remaining samples of cluster I. In general, as evidenced in Figure 2, storage conditions denoted a change in the ratio between compounds and a decrease in their intensity relative to control conditions.

Subcluster Ia included 42 of the 60 T8 samples and no T0 samples. Of these 42 samples, 29 were from orchard F and 13 from orchard S. Of the 42 samples, 14 were NINN, 11 were EINN, 10 were EIEN, and 7 were NIEN. From each 20 samples per storage condition, this subcluster included 19 samples from CA+1-MCP (A in Figure 3), 16 from DCA (B in Figure 3), and 7 from DCA+1-MCP (C in Figure 3). *trans,trans*-α-Farnesene (64–100%), along with the compounds referred for control conditions, dominated this subcluster. 2-Ethyl-1-hexanol ranged, in this case, between traces (t, < 0.05%) and 6%.

Subcluster Ib included 12 of the 20 T0 samples, 7 from orchard F and 5 from orchard S and no T8 samples. From each of the 20 samples from each growth condition, 4 were NINN, 3 were NIEN, 3 were EINN and 2 were EIEN. With shorter ranges of variation, particularly in the dominant component, *trans,trans*-α-farnesene (62–71%), the volatile profile of these samples was similar to control conditions (Table 2 and Table 3).

Subcluster Ic included just 8 T8 samples, 4 of which were from NINN, 2 from EIEN and 1 both from NIEN and EINN. Most of these samples were stored under DCA (4), and DCA+1-MCP (3) and just one from CA (Figure 3). Although still with *trans,trans*-α-farnesene (69–89%) as a major component, other T0 main components, such as hexyl acetate, were always found in trace amounts. On the other hand, 2-ethyl-1-hexanol and octanoic acid hexyl ester reached 18% and 17%, respectively, in some samples (Table 3).

Subcluster Id grouped only 6 T0 samples, 2 of which were from NINN and EIEN and 1 from both NIEN and EINN. Without 2-ethyl-1-hexanol, these were the samples with the highest range of hexyl acetate (8–16%). Despite still being dominant, *trans,trans*-α-farnesene (43–58%) showed the third lowest range in this case (Table 3).

Subcluster Ie gathered 10 samples, 2 from T0 and 8 from T8, evenly distributed between experimental conditions, with 2 to 3 per condition. 2-Ethyl-1-hexanol ranged from not detected in T0 samples to 39% in T8 samples, and pentyl hexanoate attained unusually high percentages in some cases (29%). In this subcluster, *trans,trans*-α-farnesene showed a wide range (27–71%) (Table 3).

Cluster II included just 2 T8 samples from orchard S and both from storage with ACD+1-MCP, one from NINN and the other from EIEN. These were the samples with the highest range of 2-ethyl-1-hexanol (65–71%) and the lowest range of *trans,trans*-α-farnesene (20–21%) (Table 3).

The four irrigation and fertilisation treatments (NINN, EINN, EIEN and NIEN) did not evidence a particular clustering in terms of volatiles, and they were evenly distributed in the subclusters. Instead, apple volatiles showed some tendency to group by orchard. The most evident apple volatile grouping was by harvest (T0) versus storage time (T8), and within this last condition, most samples from storage with CA+1-MCP and DCA, grouped together and separated from storage with DCA+1-MCP, as evidenced by the colour codes in Figure 3.

### 2.3. Apple Volatiles Emanation Index in T0 and T8

**Figure 4:**
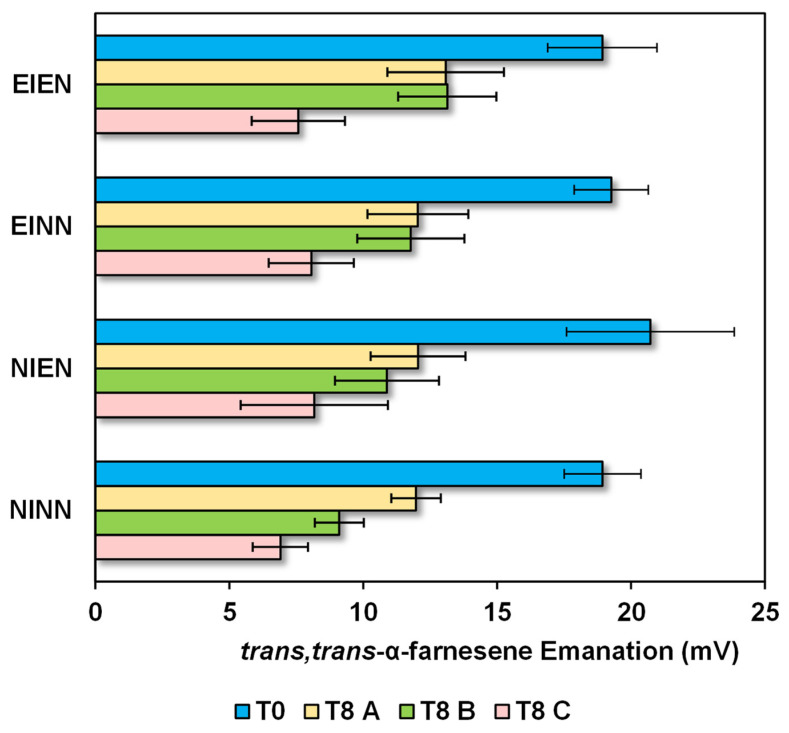
*trans,trans*-α-Farnesene emanation from the apples at T0 in the four irrigation and fertilisation treatments, NINN, EINN, EIEN and NIEN, and after 8 months (T8) under storage conditions with CA+1-MCP (A), DCA (B) or DCA+1-MCP (C).

To determine the effect of storage time and conditions on the amount of volatiles, the volatiles emanated from the apples were evaluated in a non-invasive way, as detailed in the materials and methods section. The dominant volatile component, *trans,trans*-α-farnesene, was selected, considering its response in mV in the HS-SPME-GC-FID as a measure of comparative emanation between T0 and T8 under storage conditions with CA+1-MCP, DCA, or DCA+1-MCP (A, B and C, respectively, in Figure 4).

Whatever the treatment condition, the *trans,trans*-α-farnesene emanation index ranged from 7 mV (very low) to 21 mV (extremely high). Although there was a similar range of emanation at T0 between all irrigation and fertilisation conditions (19–21 mV), apples from NIEN showed larger emanation variation (Figure 4). Overall, any of the storage conditions determined a decrease in emanation from T0 to T8, which was more accentuated in apples kept in DCA+1-MCP (Figure 4).

## 3. Discussion

There are no standard methods for fruit volatile determination. Apple volatiles, in particular, have been evaluated in either intact fruits or fruit parts, such as peel or pulp [3,26,27,37,38,39]. Since the aroma of the intact fruit is more identical to what the consumer perceives first, and the fruit break may result in the production of disruption-induced volatiles (which can also be important in taste perception), in this study the intact apples headspace solid–phase microextraction (HS–SPME) volatiles were evaluated.

As for any other plant part, fruit volatile production and release are known to be dependent on both genetic and nongenetic factors [39,40,41,42]. Apple cultivar type and maturity, cultural practices, climatic conditions, time gap between harvest and analysis, storage conditions, and volatile isolation procedures are particularly important for apple fruit volatile determination [37,39].

Previous studies on ‘Gala’ fruit volatiles either used fruit slices [12], cavities [14,15,43], juice [3] or intact fruits [16,17,18,22]. Volatile isolation was performed by vacuum steam distillation without water and recovery of compounds from a C18 column eluted with diethyl ether [12] or trapped in Tenax and desorbed in the GC [18], or in activated charcoal eluted with diethyl ether [17] or dichloromethane [16], or by solid phase microextraction of apple volatiles [22,43]. Additionally, the ‘Gala’ volatile quantification and composition was very variable. Quantification varied from µg/g [12], µmol/m^3^ [18], percentage (%) [15], intensity of response on a scale of 1 to 3 [16], µg/kg [17,44], or in thousand ion counts [22]. Composition varied from absence of reference to farnesene presence [12,16,17,18], presence in low amounts [14,15], to being the dominant compound [22]. Aroma composition and sensory parameters have also been assessed [43]. Despite this variability, which reflects the diversity of analytical conditions, compounds considered dominant in previous studies on ‘Gala’ volatiles (ex. butyl acetate, hexyl acetate, 2-methylbutyl acetate) [3,12,14,15,16,17,18,22,44] or the characteristic of a spicy aroma (estragole) [12,18,45] were also detected in the present study in variable relative amounts.

The present study established *trans,trans*-α-farnesene as the dominant volatile compound of ‘Gala’ apple intact fruits, along with high relative amounts of hexyl acetate, hexyl hexanoate, 2-methyl butyric acid hexyl ester (hexyl 2-methyl butyrate), butyl acetate, butyl hexanoate, hexyl butanoate and butyl octanoate.

This study showed that the combined effect of irrigation and nitrogen fertilisation had a lower impact on apple volatiles than the orchard origin. Although without evaluating apple volatiles, Robinson et al. [4] assessed the individual and combined effects of nitrogen fertilisation and trickle irrigation to improve ‘Gala’ fruit size and yield. The results showed that the climatic conditions of each year influence apple crop outcomes. Irrigation showed no effect on fruit size in two of the years due to above normal rainfall during that period. As rainfall was less than average in the third year, a significant improvement in fruit size and crop value with trickle irrigation was seen in that year. However, despite this positive effect, the fruit size was smaller than in the other years. The authors concluded that further environmental factors, other than soil moisture, limited fruit size in that year. Fertilisation had a significant influence on yield but no impact on fruit size.

**Figure 5:**
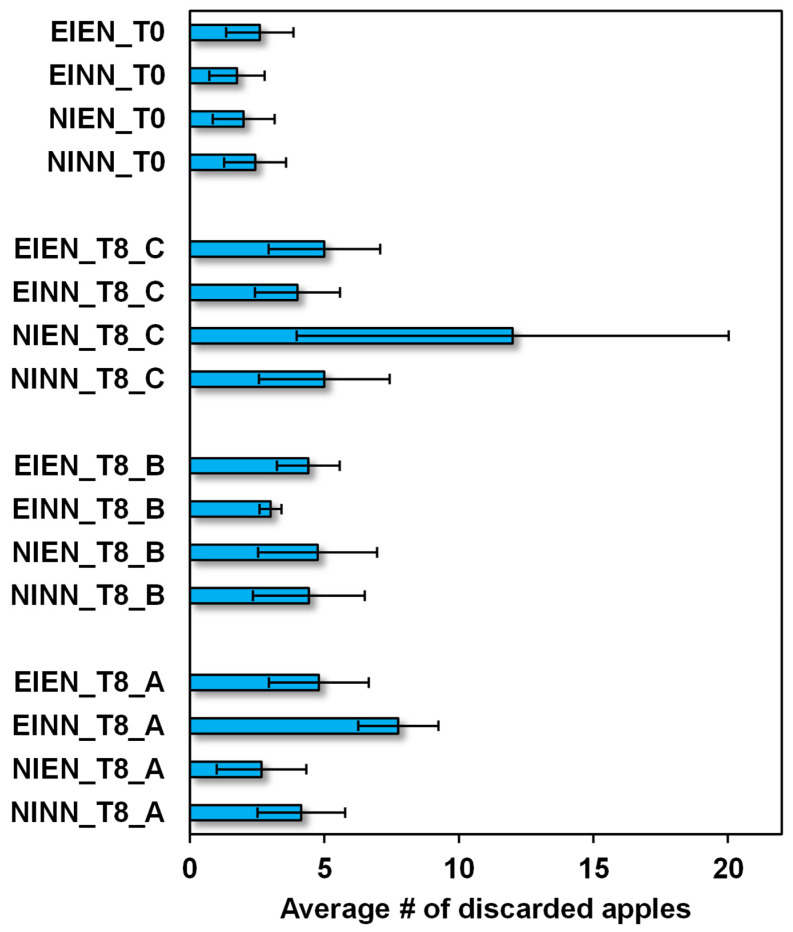
Average number (#) of discarded apples per year, in the four irrigation and fertilisation treatments, NINN, EINN, EIEN and NIEN, and after 8 months (T8) under storage conditions with CA+1-MCP (A), DCA (B) or DCA+1-MCP (C).

The storage time and storage conditions tested in the present study decreased volatile emanation and changed its chemical profile, which confirmed previous studies [3,16,17,18,23,24,46]. The less detrimental condition in terms of volatile emanation and profile was storage under CA + 1-MCP. Nevertheless, these data must be seen in combination with other apple traits, namely pulp firmness loss, sugar/acid ratio, and frequency of rot or bruises after storage (Figure 5), to find the best conditions suitable to preserve the largest set of apple attributes.

The present study is being complemented with data on sensory and quality traits analysis (fruit colour, pulp firmness, total soluble solids, titratable acidity and incidence of rot) to ascertain the right balance of storage conditions for preserving apple attributes while counteracting the negative impact on volatile emissions. The higher number of fruits with injuries and firmness loss, as well as the cost efficiency of the procedure, also questions the adequacy of some storage conditions.

## 4. Materials and Methods

### 4.1. Experimental Design

Two ‘Gala’ cultivar orchards (F and S) established at Alcobaça (mainland Portugal) were studied. The design of each apple orchard consisted of experimental blocks (trial plots), with 80 trees per treatment. The main orchard characteristics, ‘Gala’ clones, planting dates, as well as pre-harvest cultural techniques and post-harvest conservation methods are detailed in Table 1.

Pre-harvest treatments were applied by fertigation. Normal irrigation (NI) corresponds to water restitution estimated by the evapotranspiration that occurred, and the excess of irrigation (EI) is the water supply until field capacity is exceeded. An excess of nitrogen (EN) corresponds to the use of 100 units of N/ha, which is about twice the amount recommended (60 units of N/ha) (Table 1) for integrated fruit production (IFP) for orchards in production, based on the results of the foliar analysis and the expected production [47]. Both excesses were applied 1 month before harvest.

According to the fertilisation and irrigation treatment, pooled samples of fruits were collected in each orchard at each year fruit commercial harvest stage, typically by the end of August. From about 80 pooled samples of apples per condition of analysis, one-quarter (20 pooled samples) was directly used for analysis at harvest (T0), and the remaining apples were divided in similar numbers and kept under the storage conditions detailed in Table 1 for 8 months (T8). When 1-MCP was used, treatment was applied at the beginning of the storage. For this, the chambers with the fruits were hermetically closed, the temperature was set at 4 °C, and 1-MCP was applied following the manufacturer’s recommendations to a final concentration of 0.65 mg/L (Table 1). These conditions were maintained for 24 h. After this, the chambers were opened to allow air renewal, and then the process of reducing the temperature and adjusting gas concentrations began (Table 1). At T8, apples presenting any mechanical or other visible damage after storage were discarded before further assessment (Figure 5).

### 4.2. Isolation and Analysis of Apple Volatiles

For apple volatiles analysis, 14 fruits per treatment were randomly selected and divided into two aleatory sub-samples of 7 fruits, at each moment in time, and analysed separately as collective samples (Figure 1). Two replicates were performed per orchard, and conditions were assessed in each year. After harvest or after storage, the apples were kept in the dark in a cool room (16 °C) until analysis and left to stand at room temperature (24 °C) overnight, before analysis.

#### 4.2.1. Headspace Solid Phase Microextraction (HS-SPME)

‘Gala’ apple volatiles were evaluated by headspace solid phase microextraction (HS-SPME) at room temperature (Figure 1). Fruit volatiles were collected using a 100 µM polydimethylsiloxane (PDMS) coated fibre (Supelco, Bellefonte, PA, USA) inserted into a manually operated SPME holder. Each SPME fibre was thermally conditioned for up to 20 min at 250 °C before use, according to the manufacturer’s recommendations. Pooled samples of 7 intact fruits were held per glass desiccator (ø 20 cm) for 1 h for atmosphere equilibrium inside the desiccator. The fibres were then exposed in each desiccator for 1 h. Two replicas were performed per analysis. Desiccators were washed between analyses. Control experiments using SPME fibres to sample empty desiccators were carried out regularly to identify system contaminants and to confirm the absence of carry-over from repeated use of the SPME fibres.

#### 4.2.2. Analysis and Quantification of Apple Volatiles

Volatiles were analysed by Gas Chromatography with Flame Ionisation Detection (GC-FID) for quantification and by Gas Chromatography-Mass spectrometry (GC-MS) for component identification.

*Gas Chromatography*. Immediately after sampling, the SPME needle was thermally desorbed in the split/splitless injector of a Clarus 400 or a PerkinElmer AutoSystem 9000 gas chromatograph equipped with two flame ionisation detectors with a data handling system (Perkin Elmer, Shelton, CT, USA). Two columns of different polarities were inserted into the injector port: a DB-1 fused-silica column (100% dimethylpolysiloxane, 30 m × 0.25 mm i.d., film thickness 0.25 µm; Agilent, Folsom, CA, USA) and a DB-17HT fused-silica column [(50%-Phenyl)-methylpolysiloxane, 30 m × 0.25 mm i.d., film thickness 0.15 µm; J & W Scientific]. The oven temperature programme was from 45 to 175 °C, at 3 °C/min, then up to 300 °C at 15 °C/min, and finally held isothermally for 10 min, for a total run time of 61.67 min. The SPME fibres were desorbed in splitless mode for 1 min, and the gas chromatographic settings were as follows: injector and detector temperatures were 250 °C and 290 °C, respectively; carrier gas was H_2_ at 30 cm/s. The percentage composition of the apple volatiles was computed by the normalisation method (each peak reported as a percentage of the total area of all peaks detected in the data handling period) from the GC peak areas without the use of correction factors, calculated as mean values of two injections from each sample, in accordance with ISO 7609 [48].

*Gas chromatography-Mass spectrometry*. Directly after sampling, the SPME fibre was thermally desorbed in the split/splitless injector of a Perkin Elmer Clarus 690 gas chromatograph, equipped with a DB-1 fused-silica column (100% dimethylpolysiloxane, 30 m × 0.25 mm i.d., film thickness 0.25 µm; J & W Scientific), interfaced to a Perkin-Elmer SQ 8 T mass spectrometer (software version 6.1, PerkinElmer, Shelton, CT, USA). The GC conditions were the same as those used in the GC-SPME analyses described above. The transfer line temperature was 250 °C, ion source temperature, 220 °C, and the carrier gas was helium, linear velocity 30 cm/s; splitless mode for 1 min; ionisation energy, 70 eV; scan range, 40–300 amu (atomic mass unit); scan time, 1 s. Compounds were identified by calculation of their retention indices (RI) relative to a C_7_–C_17_ *n*-alkane ladder, and from mass spectra from a custom-made library based upon the analyses of reference essential oils, laboratory-synthesised components, and commercially available standards.

#### 4.2.3. Apple Volatile Emanation Index

The apple volatile emanation index was determined in a non-invasive way, considering that all compounds emitted by the sample are captured by the SPME fibre, and that all these compounds are completely desorbed in the GC-FID or in GC-MS. Considering that in each chromatogram obtained by GC-FID, the response of the dominant compound in the detector varied between 5 and 25 mV, the volatile emanation index was evaluated as extra-high (6) with a response >16.00 mV, very high (5) with a response between 14.90 and 16.00 mV, high (4) between 12.70 and 14.89 mV, moderate (3) between 9.40 and 12.60 mV, low (2) between 7.20–9.39 mV and very low if <7.20 mV.

#### 4.2.4. Statistical Analysis

The percent composition of apple volatiles was used to determine the relationship between the different samples by cluster analysis using the Numerical Taxonomy Multivariate Analysis System (NTSYS PC software, version 2.2, Exeter Software, Exeter University, Exeter, UK) [49]. For cluster analysis, the correlation coefficient was selected as a measure of similarity among all samples, and the Unweighted Pair Group Method with Arithmetical Averages (UPGMA) was used for cluster definition. The degree of correlation was evaluated according to Pestana and Gageiro [50] as very high (0.90–1.0), high (0.70–0.89), moderate (0.40–0.69), low (0.20–0.39), and very low (<0.20).

## 5. Conclusions

To the best of our knowledge, this study details for the first time the volatile profile of “Maçã de Alcobaça” cv. ‘Gala’ and the effect of cultural practices and conservation method on their volatile composition and intensity.

This study showed that apple volatile variability is more dependent on each orchard’s conditions, such as edaphoclimatic conditions, and the apple clone than on irrigation and nitrogen fertilisation amount. On the other hand, storage time and storage conditions were shown to determine not only a lower emanation rate but also a change in the volatiles profile.

To what extent the orchard age, apple clone, rootstock and cultural practices, such as pruning, may also influence the volatile variability per orchard should be further explored.

## Figures and Tables

**Table 1:** Summary of the experimental setup. Orchards, ‘Gala’ clones, rootstock, orchard planting dates, irrigation and fertilisation treatments, conditions used during the 8 months of storage and total number of samples analysed in the four year study (2018–2021) per orchard.

Orchard				F *	S *
**Clone**				Venus Fengal	Schniga Schnico
**Rootstock**				M9	M9
**Planting date**				2015	2014
**Irrigation/Fertilisation treatment**	**Code**		**Kg/ha**	Total # Samples **	
Regular irrigation and nitrogen fertilisation (Control)	NINN	N	60	16	12
		H_2_O	EE		
Regular irrigation and excess nitrogen	NIEN	N	100	4	11
		H_2_O	EE		
Excess irrigation and regular nitrogen	EINN	N	60	16	1
		H_2_O	EFC		
Excess irrigation and nitrogen fertilisation	EIEN	N	100	12	8
		H_2_O	EFC		
**Storage conditions (8 months)**		**°C**	**O_2_ %**	**CO_2_ %**	**RH %**
Controlled Atmosphere + 1-methylcyclopropene (CA + 1-MCP ***)	A	1	2	2	90
Dynamic Controlled Atmosphere (DCA) ****	B	1	0.5	1	95
Dynamic Controlled Atmosphere + 1-Methylcyclopropene (DCA + 1-MCP)	C	1	0.5	1	95

* To ensure data protection each orchard was assigned with an arbitrary code letter. ** Total number of pooled samples in the four year study per orchard, including at harvest (T0) and 8 months after storage (T8). #: Number. EE: water amount equivalent to the estimated evapotranspiration. EFC: water amount exceeds field capacity. RH: Relative humidity. *** 1-MCP: 0.65 mg/l. **** The protocol adopted for DCA chambers started with the reduction of the O_2_ level to 0.5% in 24–30 h. Later, the percentage of O_2_ was adjusted and kept at 0.45–0.5%.

**Table 2:** Minimum and maximum percentage range composition of the volatiles isolated by Headspace Solid Phase Microextraction (HS-SPME) from *Malus domestica* cv. ‘Gala’ (“Maçã de Alcobaça”) fruits at harvest time (T0) from the plots grown under control conditions, with regular irrigation and nitrogen fertilisation (NINN).

	HS-SPME-Isolated Volatiles in Control Condition at T0			
Peak #	Components	RI	Min	Max
1	isoamyl alcohol	722	t	0.4
2	butyl acetate	854	1.5	6.2
3	*n*-hexanol	881	t	0.6
4	2-methyl butyl acetate	882	0.9	3.4
5	propionic acid butyl ester (=butyl propionate)	899	t	0.4
6	amyl acetate	900	t	0.3
7	butyl butyrate	973	0.6	1.8
8	hexyl acetate	995	5.2	23.0
9	2-methyl butyric acid butyl ester (=2-methylbutyl 2-methylbutyrate)	1017	0.5	1.5
10	isoamyl butanoate	1030		t
11	amyl butanoate	1066	t	0.3
12	propionic acid hexyl ester (=hexyl propionate)	1079	0.5	1.1
13	butyl tiglate	1105		t
14	2-methyl butyric acid pentyl ester (=pentyl 2-methylbutyrate)	1116	t	0.1
15	hexyl isobutanoate	1127		0.2
16	2-ethyl hexyl ester	1144	t	0.4
17	methyl chavicol (=estragole)	1163	t	1.2
18	butyl hexanoate	1173	1.8	5.7
19	hexyl butanoate	1173	2.1	5.7
20	octyl acetate (=octanol acetate)	1189		0.1
21	2-methyl butyric acid hexyl ester (=hexyl 2-methyl butyrate)	1220	2.9	6.1
22	isoamyl hexanoate	1240	t	1.0
23	pentyl hexanoate	1270	t	0.3
24	butyl heptanoate	1271	t	0.3
25	hexyl tiglate	1316	t	0.2
26	decanoic acid	1356	t	0.1
27	*n*-undecanol	1366	t	0.1
28	hexyl hexanoate	1375	3.0	9.3
29	butyl octanoate	1376	2.2	5.8
30	*n*-tetradecane	1400	t	0.4
31	isoamyl octanoate	1436	t	0.7
32	*trans*-β-farnesene	1455	t	0.2
33	Amyl octanoate	1472	t	0.1
34	*cis,trans*-α-farnesene *	1484	t	0.5
35	*trans,trans*-α-farnesene	1500	35.8	69.1
36	*n*-pentadecane	1500		t
37	octanoic acid hexyl ester (=hexyl octanoate, hexyl caprylate)	1559	0.2	0.9
38	butyl decanoate	1563	t	0.1
39	*n*-hexadecane	1600	t	t
40	*trans,cis*-farnesol	1648	t	0.3
	**% of identification**		97.4	99.7
	**Grouped components**			
	Sesquiterpene hydrocarbons		35.8	69.6
	Oxygen-containing sesquiterpenes		t	0.3
	Phenylpropanoids		t	1.2
	Fatty acids		t	0.1
	Alkanes		t	0.4
	Others		28.0	63.9

#: Number. RI: In lab calculated Retention Index relative to C_7_–C_17_ *n*-alkanes on the DB-1 column. * Identification based on mass spectra only. t: trace (< 0.05%).

**Table 3:** Minimum and maximum percentage range of HS-SPME-isolated volatiles from intact *Malus domestica* cv. Gala (“Maçã de Alcobaça”) from all treatments and from T0 and T8. For samples grouped by each of the clusters and subclusters, *vide* Figure 3.

		HS-SPME-Isolated Volatiles in All Growth and Storage Conditions											
		Cluster I										Cluster II	
		Ia		Ib		Ic		Id		Ie			
Components	RI	Min	Max	Min	Max	Min	Max	Min	Max	Min	Max	Min	Max
isoamyl alcohol	722	t	0.1	t	0.4	t	t	t	0.5	t	0.7	t	t
butyl acetate	854	t	t	0.7	3.2	t	t	0.9	3.2	t	6.2	t	t
*n*-hexanol	881	t	t	t	0.2	t	t	t	1.1	t	1.4	t	t
2-methyl butyl acetate	882	t	0.2	0.8	2.0	t	t	0.9	2.4	t	3.4	t	t
propionic acid butyl ester	899	t	1.9	t	0.5	t	t	t	0.2	t	5.9	t	t
amyl acetate	900	t	0.4	t	0.2	t	t	t	0.3	t	0.5	t	t
butyl butyrate	973	t	0.5	0.4	0.8	t	t	0.6	1.1	t	1.8	t	t
hexyl acetate	995	t	2.5	4.3	7.3	t	t	7.6	15.9	t	23.3	t	t
2-ethyl-1-hexanol	1004	t	6.1			t	17.7				39.4	65.0	71.0
2-methyl butyric acid butyl ester	1017	t	0.4	0.8	1.5	t	t	0.3	0.8	t	0.6	t	t
isoamyl butanoate	1030	t	t	t	t	t	t	t	t	t	t	t	t
amyl butanoate	1066	t	0.4	0.1	0.3	t	0.2	t	0.1	t	1.5	t	1.7
propionic acid hexyl ester	1079	t	0.7	0.5	1.1	t	t	0.2	0.7	t	6.9	t	t
butyl tiglate	1105				0.1			t	0.1		t		
2-methyl butyric acid pentyl ester	1116	t	t	t	0.1	t	t	t	0.1	t	t	t	t
hexyl isobutanoate	1127	t	1.6	0.1	0.2	t	t	0.2	0.2	t	t	t	t
2-ethyl hexyl ester	1144	t	5.3	t	0.4	t	6.6	t	0.6	t	13.8	t	t
methyl chavicol	1163	t	2.3	t	1.2	t	t	t	0.2	t	0.3	t	t
butyl hexanoate	1173	t	4.1	1.8	4.2	t	1.7	3.7	4.3	t	5.7	1.8	4.7
hexyl butanoate	1173	t	4.1	2.1	4.2	t	1.7	4.1	7.2	t	5.7	1.8	4.7
octyl acetate	1189		t	t		t	t		t		t		
2-methyl butyric acid hexyl ester	1220	t	3.2	2.6	6.1	t	0.6	4.2	7.0	t	7.2	t	t
isoamyl hexanoate	1240	t	1.6	0.1	1.0	t	0.3	t	0.1	t	t	t	t
pentyl hexanoate	1270	t	2.0	0.2	0.3	t	t	0.2	0.3	t	28.6	t	t
butyl heptanoate	1271	t	0.5	0.2	0.3	t	t	0.2	0.3	t	0.2	t	t
hexyl tiglate	1316	t	0.2	0.1	0.2	t	t	t	0.2	t	0.2	t	t
decanoic acid	1356	t	0.3	t	0.1	t	t	t	0.1	t	t	t	t
*n*-undecanol	1366	t	0.3	t	0.1	t	t	t	0.1	t	t	t	t
hexyl hexanoate	1375	t	8.1	2.9	5.0	t	7.3	5.3	9.3	t	9.4	t	t
butyl octanoate	1376	t	5.8	1.8	3.2	t	7.3	3.7	5.8	t	6.2	t	t
*n*-tetradecane	1400	t	0.3	t	0.5	t	t	0.1	0.4	t	0.2	t	t
isoamyl octanoate	1436	t	1.4	t	0.7	t	0.4	t	0.2	t	t	t	t
*trans*-β-farnesene	1455	t	1.3	t	0.2	t	0.3	t	0.1	t	0.1	t	t
amyl octanoate	1472	t	0.2	t	0.1	t	t	0.1	0.1	t	0.1	t	t
*cis,trans*-α-farnesene *	1484	t	1.0	0.2	0.5	t	0.8	0.1	0.3	t	0.2	t	t
*trans,trans*-α-farnesene	1500	64.1	100.0	62.3	70.7	68.5	88.8	43.0	58.1	27.9	71.4	19.9	21.3
*n*-pentadecane	1500	t	0.3		t		t		t		t	t	t
octanoic acid hexyl ester	1559	t	3.2	0.2	0.3	t	16.8	0.3	0.9	t	25.7	t	t
butyl decanoate	1563	t	0.2	t	0.1	t	t	t	0.1	t	0.1	t	t
*n*-hexadecane	1600	t	0.2	t	0.1	t	t	t	t	t	t	t	t
*trans,cis*-farnesol	1648	t	0.7	t	0.2	t	t	0.1	0.3	t	0.3	t	t
**% of identification**		95.0	100.0	96.9	99.6	95.8	99.9	97.3	99.1	92.4	100.0	95.7	96.2
**Grouped components**													
Sesquiterpene hydrocarbons		64.8	100.0	62.6	71.0	68.5	88.8	43.3	58.3	27.9	71.4	19.9	21.3
Oxygen-containing sesquiterpenes		t	0.7	t	0.2	t	t	0.1	0.3	t	0.3	t	t
Phenylpropanoids		t	2.3	t	1.2	t	t	t	0.2	t	0.3	t	t
Fatty acids		t	0.3	t	0.1	t	t	t	0.1	t	t	t	t
Alkanes		t	0.6	t	0.6	t	t	0.1	0.4	t	0.2	t	t
Others		t	28.2	25.5	35.2	10.4	31.4	40.0	55.1	27.7	69.8	74.4	76.3

RI: In-lab calculated retention index relative to C_7_–C_17_ *n*-alkanes on the DB-1 column. * Identification based on mass spectra only. t: trace (<0.05%).

## Data Availability

Not applicable.

## Sample Availability

Samples are available from the authors.
